# Expression of hereditary hemochromatosis C282Y HFE protein in HEK293 cells activates specific endoplasmic reticulum stress responses

**DOI:** 10.1186/1471-2121-8-30

**Published:** 2007-07-24

**Authors:** Matthew W Lawless, Arun K Mankan, Mary White, Michael J O'Dwyer, Suzanne Norris

**Affiliations:** 1 Hepatology Research Division and Department of Clinical Medicine, Institute of Molecular Medicine, Trinity Centre for Health Sciences, Trinity College Dublin, St. James Hospital, Dublin, Ireland

## Abstract

**Background:**

Hereditary Hemochromatosis (HH) is a genetic disease associated with iron overload, in which individuals homozygous for the mutant C282Y *HFE *associated allele are at risk for the development of a range of disorders particularly liver disease. Conformational diseases are a class of disorders associated with the expression of misfolded protein. HFE C282Y is a mutant protein that does not fold correctly and consequently is retained in the Endoplasmic Reticulum (ER). In this context, we sought to identify ER stress signals associated with mutant C282Y HFE protein expression, which may have a role in the molecular pathogenesis of HH.

**Results:**

Vector constructs of Wild type HFE and Mutant C282Y HFE were made and transfected into HEK293 cell lines. We have shown that expression of C282Y HFE protein triggers both an unfolded protein response (UPR), as revealed by the increased GRP78, ATF6 and CHOP expression, and an ER overload response (EOR), as indicated by NF-κB activation. Furthermore, C282Y HFE protein induced apoptotic responses associated with activation of ER stress. Inhibition studies demonstrated that tauroursodeoxycholic acid, an endogenous bile acid, downregulates these events. Finally, we found that the co-existence of both C282Y HFE and Z alpha 1-antitrypsin protein (the protein associated with the liver disease of Z alpha 1-antitrypsin deficiency) expression on ER stress responses acted as potential disease modifiers with respect to each other.

**Conclusion:**

Our novel observations suggest that both the ER overload response (EOR) and the unfolded protein response (UPR) are activated by mutant C282Y HFE protein.

## Background

Hereditary Hemochromatosis (HH) is a disorder of iron metabolism, associated with accumulation of iron in the body. HH is an autosomal recessive disorder resulting from mutations in the *HFE *(hemochromatosis) gene, with clinical consequences which can range from cirrhosis of the liver, diabetes, heart failure, arthritis to liver cancer [1,2]. The protein encoded by this gene is similar to MHC class I-type proteins and associates with β_2_-microglobulin (β_2_M). β_2_M has been shown to play an important role in regulating iron absorption. The majority of HH patients carry an *HFE *mutation, in which cysteine at position 282 is changed to tyrosine (C282Y) [3,4]. This mutation prevents the formation of a disulfide bond in the α3 domain, impairing the normal association of HFE with β_2_M. This lack of association dramatically reduces the cell surface expression of HFE, whereby the majority of the C282Y mutant protein remains as high molecular weight aggregates, fails to undergo late Golgi processing and subsequently is retained in the endoplasmic reticulum (ER) [3,5-7]. This retention of the mutant protein in the ER may provide a basis for the pathogenesis of this condition. In line with this, we propose that HH associated with C282Y HFE, may be viewed in the context of conformational diseases.

Conformational diseases arise from changes that lead to aggregation and retention of protein with consequent late or episodic onset of symptoms [8]. Cells respond to this perturbation in the ER by inducing the expression of novel genes whose products may restore ER function [9] but may also act as proinflammatory stimulants. Regulation of these ER stress genes occurs *via *two signal transduction pathways: the ER overload response (EOR) and the unfolded protein response (UPR). The EOR activates IKK (inhibitor of κB kinase), which causes degradation of the inhibitor of κB (I-κB). This allows for the activation of NF-κB, a transcription factor that induces the expression of genes encoding several proteins involved in death, survival and inflammatory responses [10]. The UPR pathway involves up-regulation of glucose responsive genes such as grp78 (Ig H chain binding protein (BiP)) in order to facilitate protein trafficking in the ER and the transcription factor CHOP/GADD153 (CCAAT/enhancer-binding protein (C/EBP)) [11-13], which sensitizes cells to ER stress. In this regard, ATF-6 is a member of the ATF/CREB basic-leucine zipper (bZIP) and a proximal transducer of both GRP78/BiP and CHOP/GADD153 [14,15]. These protective responses are designed to relieve ER stress, however when they are ineffective, the cell undergoes apoptosis [16]. Bcl-2 is an antiapoptotic protein located in the membranes of the ER [17]. Studies have shown that ER stress downregulates Bcl-2, culminating in cytochrome *c *release and activation of caspase-3 [18,19]. Tauroursodeoxycholic acid (TUDCA), an endogenous bile acid synthesized in the liver's conjugation pathway of urodeoxycholic acid (UDCA), has been demonstrated to inhibit these ER stress-induced pathways [20-22]. It is also reported to have a role in preventing toxicity by reducing inflammatory signals [23]

The mechanisms involved in the intracellular processing of mutant proteins are complex and not fully understood [16,24]. Z α-_1_antitrypsin (A1AT) deficiency is a disorder involving a polymerogenic mutant of the secretory glycoprotein A1AT. The protein product of the mutant Z A1AT gene is synthesized in hepatocytes but accumulates intracellularly rather than being secreted from the cell. The downstream effects of the expression of the mutant Z protein include ER stress resulting in mitochondrial injury and caspase activation [25-27]. Chronic liver disease develops in a subgroup of Z A1AT deficient individuals associated with the mutant Z A1AT protein [28-30]. Currently, liver transplantation is the only treatment available for severe liver disease associated with Z A1AT deficient individuals [25,28]. The variable clinical presentations among affected individuals, suggests an important contribution of genetic and environmental disease modifiers. While early studies have reported an association between HH and the Z A1AT mutation which may modify the onset and severity of the liver disorder [31,32], to date this possible association from clinical population studies remains unclear [33]. Indeed, molecular studies have shown that activation of the Z A1AT UPR pathway only occurs in the presence of a secondary stimulus. This lack of activation of the UPR pathway has been suggested as an explanation for the onset of the liver disorder Z A1AT deficiency [34].

While C282Y HFE HH is considered a monogenic disease its phenotypic expression varies considerably [4]. We propose that differences in the quality control systems within the ER could explain some of this phenotypic variation. In this study, we test this hypothesis and demonstrate that ER stress is associated with mutant C282Y HFE expression. We determine the potential usefulness of TUDCA on C282Y HFE induced ER stress [20-22]. Furthermore, the co-existence of both C282Y HFE and Z A1AT protein expression on ER stress responses were investigated (in particular C282Y HFE ability to activate the ZA1AT UPR pathway) as potential disease modifiers with respect to each other.

## Results

### C282Y HFE model system

**Figure 1:**
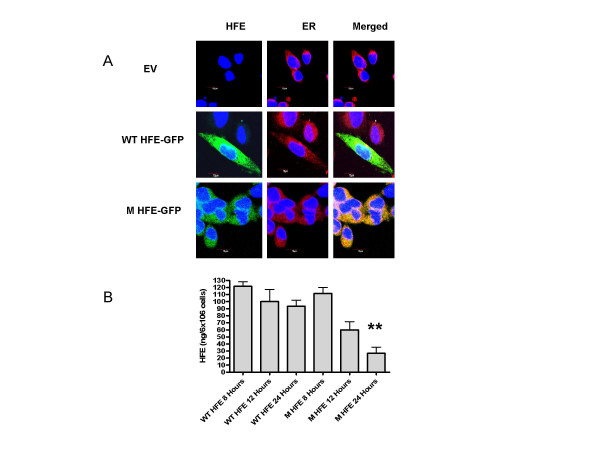
**Intracellular localization of WT HFE and C282Y HFE in HEK293 transfected cells**. (A) confocal microscopy of cells cultured for 24 h after transfection with EV-GFP, WT-HFE GFP and C282Y-HFE GFP stained with an antibody for the ER-resident protein calnexin and probed with Texas Red labeled secondary antibody. In the merged images, the yellow color corresponds to areas in which the green and red stainings overlap. The nucleus appears blue due to DNA staining with 4',6-diamidino-2-phenylindole. Scale bar, 10 μm. (B) HFE expression in transfected HEK293 cell lysates. HEK 293 cells (6 × 10^6^) were transfected with pcDNA3.1 (empty vector, EV), WT HFE, or C282Y HFE (M) expression plasmids. C282Y HFE-His tag protein production in lysates was measured by ELISA. C282Y HFE-His tag-transfected cells showed markedly accelerated degradation of the C282Y HFE protein compared to N HFE protein (**, p = 0.0012) at 24 hours compared to WT HFE expression. Assays were performed in triplicate.

To confirm C282Y HFE accumulated within the ER, we performed confocal microscopy on 24-h post transfected cells using GFP tagged HFE protein along with antibodies against the ER resident protein calnexin. The C282Y HFE co-localized with calnexin (Figure. 1A), as illustrated by merged images of the overlapping C282Y HFE/calnexin which appears yellow, whereas the WT HFE did not co-localize remaining green. Degradation of C282Y HFE was found compared to WT HFE protein by GFP intensity. This was confirmed with lysates from C282Y HFE-His tag-transfected cells which showed degradation of the C282Y HFE protein compared to WT HFE protein (p = 0.0012) at 24 hours (Figure. 1B).

### C282Y activation of the EOR pathway

**Figure 2:**
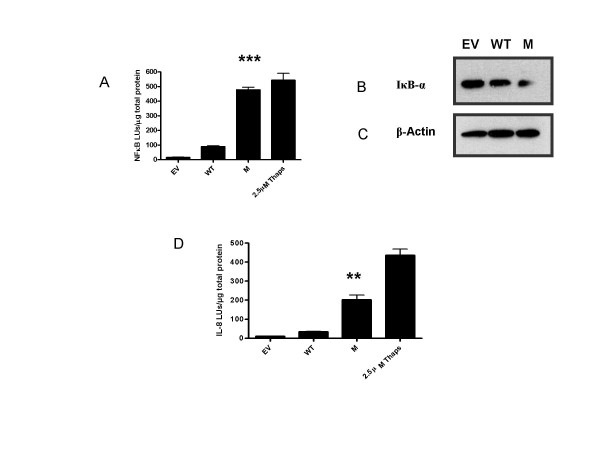
**C282Y HFE activates EOR**. (*A*) NF-κB-luciferase measurement. HEK 293 cells (1 × 10^6^) were transfected with pcDNA3.1 (empty vector, EV), WT HFE, or C282Y HFE (M) expression plasmids and an NF-κB-luciferase reporter gene vector. As positive control, five hours post transfection cells transfected with NF-κB-luciferase reporter gene vector were stimulated with 2.5 μM thapsigargin for 24 h. Cells were incubated for 24 h in serum-complete medium. Luciferase production in cell lysates was measured by luminometery. Levels are expressed as light units (LU) per microgram of total protein (***, *p *= 0.0009) compared to WT HFE. Assays were performed in triplicate and are representative of at least three separate experiments. (*B*) Degradation of IκB-α by C282Y HFE. HEK 293 cells were transfected with pcDNA3.1 (empty vector, EV), WT HFE, or C282Y HFE expression plasmids, and cytosolic extracts were prepared 24 h post-transfection. Ten-microgram of protein was assayed for IκB-α degradation by Western blotting (*n *= 3). (*D*) HEK 293 cells (1 × 10^6^) were transfected with pcDNA3.1 (empty vector, EV), WT HFE, or C282Y HFE expression plasmids and an IL-8-luciferase reporter gene vector. As positive control, five hours post transfection cells transfected with IL-8-luciferase reporter gene vector were stimulated with 2.5 μM thapsigargin for 24 h. Cells were incubated for 24 h in serum-complete medium. Luciferase production in cell lysates was measured by luminometry. Levels are expressed as light units (LU) per microgram of total protein of total protein (**, *p *= 0.0031) compared to WT HFE. Assays were performed in triplicate and are representative of at least three separate experiments.

To examine the effects of C282Y HFE on the EOR pathway, activation of NF-κB by C282Y HFE was measured by NF-κB luciferase reporter gene assay (Fig. 2A). NF-κB promoter activation was measured in Empty vector (EV), WT HFE-transfected, C282Y HFE-transfected and control cells stimulated with 2.5 μM thapsigargin, 24 h post-transfection using an NF-κB-linked luciferase reporter gene. Time course studies demonstrated that treatment for 24 h induced optimal NF-κB promoter activity (data not shown). C282Y HFE induced significantly higher levels of luciferase expression compared to control or WT HFE-transfected cells (*p *= 0.0009) suggesting that overexpression of C282Y HFE leads to activation of NF-κB. We further confirmed NF-κB activation by analyzing IκB-α degradation in the same cells, by western immunoblotting. IκB-α, 24 h post-transfection, was degraded in C282Y HFE-transfected cells compared with control or WT HFE-transfected cells (Fig. 2B). Blots were stripped and reprobed using a monoclonal antibody to β-Actin to confirm equal loading (Fig. 2C).

To further elucidate these signaling events, IL-8 promoter activity was measured using an IL-8 promoter luciferase reporter gene in EV-transfected, WT HFE-transfected, C282Y HFE-transfected and control cells stimulated with 2.5 μM thapsigargin. As NF-κB activation regulates the transcription of the proinflammatory target gene IL-8, activation of IL-8 further confirmed induction of NF-κB. C282Y HFE induced significantly higher levels of luciferase activity compared with those in WT HFE-transfected cells (*p *= 0.0031) suggesting that overexpression of C282Y HFE leads to activation of IL-8 (Fig. 2D).

### C282Y HFE activates the UPR pathway and is a secondary stimulus activating Z A1AT UPR

**Figure 3:**
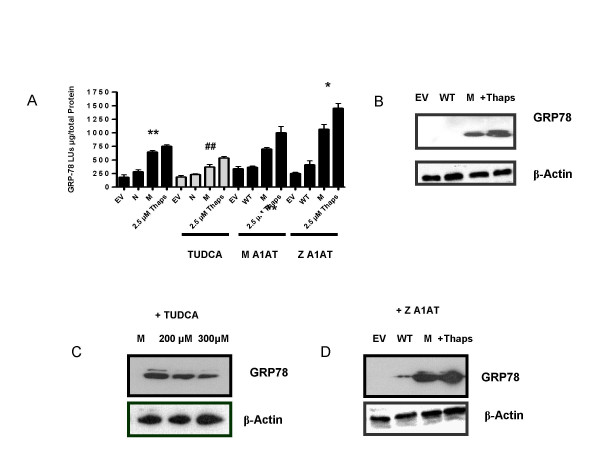
**Activation of the UPR by C282Y HFE**. (A) HEK 293 cells were transfected with pcDNA3.1 (empty vector, EV), WT HFE, or C282Y HFE (M) expression plasmids and a grp78-promoter luciferase reporter gene vector (*grp78*-luc) with or without pZeoSv2^+ ^(data not shown), pMA1AT, or pZA1AT. As positive control or treatment, five hours post transfection cells were incubated with 2.5 μM thapsigargin or 300 μM TUDCA acid for 24 h. Cells were incubated for 24 h in serum-complete medium. Luciferase production in cell lysates was measured by luminometry. Levels are expressed as light units (LU) per microgram of total protein. C282Y HFE activated *grp78*-luc expression (**, p = 0.0011) compared to WT HFE. Treatment with TUDCA significantly decreased grp78-LUC expression (##, p = 0.0006) compared to WT HFE. Z A1AT transfection significantly increased grp78-LUC expression (*, p = 0.016) compared to N HFE. Assays were performed in triplicate and are representative of at least three separate experiments. (B) GRP78/BIP protein production was analyzed by western blot in cytosolic extracts (10 μg) from HEK293 cells transfected with pcDNA3.1 (lane 1), WT HFE (lane 2), C282Y HFE (lane 3) or HEK293 cells stimulated with 2.5 μM thapsigargin (lane 4; n = 3). (C) GRP78/BIP protein production were analyzed by western blot in cytosolic extracts (10 μg) from HEK293 cells transfected with C282Y HFE untreated (lane 1) treated with 200 μM TUDCA (lane 2) 300 μM TUDCA (lane 3). (D) GRP78/BIP protein production were analyzed by western blot in cytosolic extracts (10 μg) from HEK293 cells transfected with pZA1AT with pcDNA3.1 (lane 1), WT HFE (lane 2) or C282Y HFE (lane 3) or HEK293 cells stimulated with 2.5 μM thapsigargin (lane 4; n = 3). Blots were stripped and probed for β-Actin to confirm equal loading.

We examined the effects of C282Y HFE expression on UPR activation by investigating its effect on the *grp78 *promoter and GRP78/BiP protein production. C282Y HFE resulted in increased expression of a *grp78*-promoter-linked luciferase reporter gene compared to WT HFE-transfected cells (** *p *= 0.0011) (Fig. 3A). We confirmed this by measuring GRP78/BiP protein production by western immunoblotting. We found that GRP78/BiP protein production is increased in C282Y HFE-transfected cells compared with EV or WT HFE-transfected cells (*n *= 3) (Fig. 3B). Thapsigargin, a known ER agonist used as a positive control, increased *grp78 *promoter-linked luciferase reporter gene and protein expression in cells. Blots were stripped and probed for β-Actin in all these experiments. We next examined the effect of TUDCA as an inhibitor of C282Y HFE UPR activation. We investigated its effect on the *grp78 *promoter and GRP78/BiP protein expression. TUDCA significantly decreased levels of luciferase expression in C282Y HFE-transfected cells treated with 300 μM TUDCA compared with those without treatment (##, *p *= 0.006) (Fig. 3A). Time course studies demonstrated that a dose of 300 μM TUDCA showed decreased *grp78 *promoter activity (data not shown). We further confirmed TUDCA's potential effect by analyzing GRP78/BiP protein production in the same cells by western immunoblotting. 24 h post-transfection, cells treated with 200 μM and 300 μM TUDCA had significantly decreased levels of GRP78/BiP protein production compared to those without TUDCA treatment (*n *= 3) (Fig. 3C).

To measure the effect of C282Y HFE expression on Z A1AT UPR activation, *grp*78 promoter and GRP78/BiP protein production was measured. We found that C282Y HFE protein increases activation of *grp78 *expression in the Z A1AT cell line compared to M A1AT (*, *p *= 0.016) (Fig. 3A). WT HFE did not induce Z A1AT *grp78 *promoter activation nor did Z A1AT (without C282Y HFE) (Data not shown) (34). To confirm these findings we examined GRP78/BiP protein production by western immunoblotting. Fig. 3D shows that GRP78/BiP protein production is increased in the Z A1AT transfected cell line compared with EV or M (wild type) A1AT-transfected cells. Thapsigargin-treated cells were used as a positive control (Fig. 3D).

### C282Y HFE activates ATF-6 and CHOP components of the UPR pathway and is a secondary stimulus activating the Z A1AT UPR pathway

**Figure 4:**
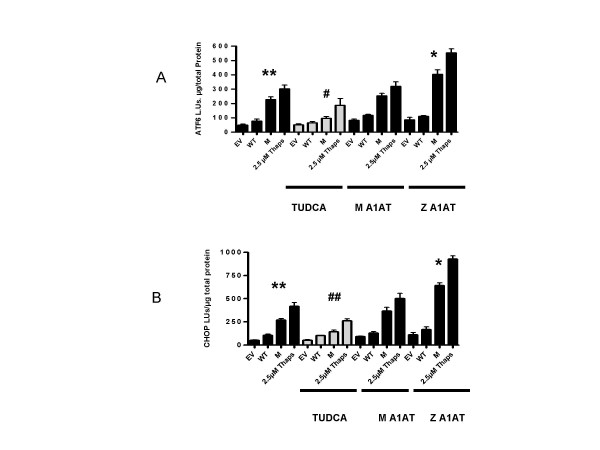
**Activation of the ATF-6, CHOP by C282Y HFE**. HEK 293 cells were cotransfected with pcDNA3.1 (empty vector, EV), WT HFE, or C282Y HFE (M) expression plasmids and (A) ATF6-promoter luciferase reporter gene vector (ATF6-luc) (B) CHOP-promoter luciferase reporter gene vector (CHOP-luc), with or without pZeoSv2^+ ^(data not shown). As positive control or treatment, five hours post transfection cells were incubated with 2.5 μM thapsigargin or 300 μM TUDCA for 24 h. Cells were incubated for 24 h in serum-complete medium. Luciferase production in cell lysates was measured by luminometry. Levels are expressed as light units (LU) per microgram of total protein. C282Y HFE activated ATF-6 and CHOP-luc expression (**, p = 0.0039 and **, p = 0.0011 respectively) compared to WT HFE. Treatment with TUDCA significantly decreased ATF6-luc and CHOP-luc expression (#, p = 0.0175 and ##, p = 0.0067 respectively) compared to WT HFE. Z A1AT transfection significantly increased ATF6-luc and CHOP-luc expression (*, p = 0.02 and **, p = 0.0059) compared to WT HFE. Assays were performed in triplicate and are representative of at least three separate experiments.

We examined C282Y HFE expression on other components of the UPR pathway. ATF-6 has direct transcriptional activating properties for UPR target genes, including the transcription factor CHOP and *grp78 *itself. We found that C282Y HFE transfected cells resulted in increased expression of a *ATF6 *and *CHOP*-promoter-linked luciferase reporter gene compared to WT HFE cells (Fig. 4A and 4B) (** *p *= 0.0039 and ** *p *= 0.0011). TUDCA significantly decreased levels of *ATF6 *and *CHOP *luciferase expression in C282Y HFE-transfected cells treated with 300 μM TUDCA compared to untreated (Fig. 4A and 4B) (# *p *= 0.0175 and ## *p *= 0.0067 respectively). C282Y HFE protein expression significantly increased *ATF-6 *and *CHOP *promoter activity in the Z A1AT cell line compared to M A1AT (*, *p *= 0.02 and **, *p *= 0.0059, respectively) (Fig. 4A and 4B). WT HFE did not induce Z A1AT *ATF-6 *and *CHOP *promoter activation nor did Z A1AT without C282Y HFE protein in control experiments (Data not shown).

### C282Y HFE induces an apoptotic response

**Figure 5:**
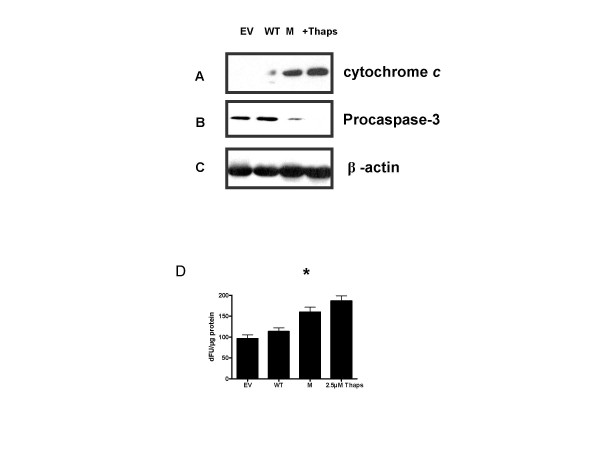
**C282Y HFE induces cytochrome *c *release and caspase-3 activation**. HEK293 cells (1 × 10^6^) were transfected with EV, WT HFE, C282Y HFE expression plasmids or untransfected cells treated with 2.5 μM thapsigargin, and cytosolic extracts were prepared 24 h posttransfection. Ten-microgram of protein was assayed for cytochrome *c *release (A), and caspase-3 activation (B) by western blotting (n = 3). C282Y HFE (M) induced cytochrome *c *release and processing of procaspase-3 compared to EV, WT HFE or 2.5 μM thapsigargin treated cells. (C) To confirm equal loading blots were stripped and probed for β-Actin. (D) Measurement of caspase-3 activity in the lysates of HEK293 cells (1 × 10^6^) transfected with control (empty vector), WT HFE or C282Y HFE expression vectors, and positive control of 2.5 μM thapsigargin was used. C282Y HFE significantly increased caspase-3 activity (*, p = 0.0363) (n = 3).

We next examined whether C282Y HFE activation of the UPR pathway induced cell death responses. In order to examine potential C282Y HFE induced apoptotic responses associated with end stage ER stress, we investigated its effects on key ER stress cell death mediators. Cytochrome *c *release was examined by western immunoblotting. We found increased cytochrome *c *release in C282Y HFE transfected cells compared to WT HFE or EV (Fig. 5A). We confirmed this response by probing for caspase-3 activation. C282Y HFE transfected cells had enhanced activation of caspase-3 compared to WT HFE or EV (Fig. 5B). Equal loading was confirmed by stripping the blots and probing with an antibody to β-Actin (Fig. 5C). To confirm this finding, we examined the effect of C282Y HFE on caspase-3 activity. HEK293 cells were transfected with EV, WT HFE or C282Y HFE expression plasmids. Thapsigargin was used as a positive control. Caspase-3 activity was measured using a fluorometric caspase-3 substrate. C282Y but not WT HFE induced caspase-3 activity significantly compared to EV (*, p = 0.0363) (Fig. 5D).

### Tauroursodeoxycholic acid inhibits C282Y HFE induced apoptotic responses

**Figure 6:**
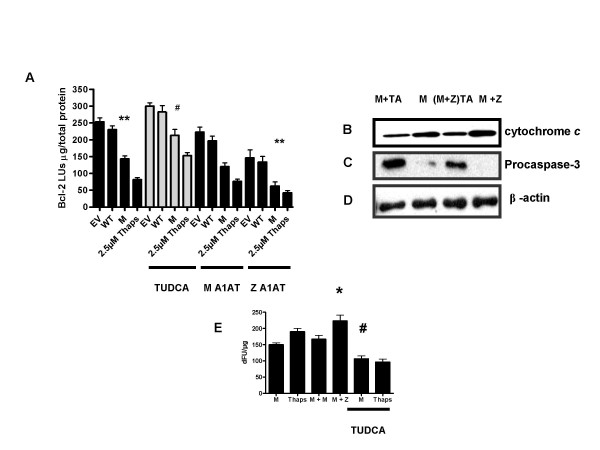
**Taurosodeoxycholic acid inhibits C282Y HFE induced apoptotic responses**. (*A*) HEK 293 cells (1 × 10^6^) were transfected with pcDNA3.1 (empty vector, EV), WT HFE, or C282Y HFE (M) expression plasmids and an *bcl-2*-luciferase reporter gene vector. As positive control, five hours post transfection cells were stimulated with 2.5 μM thapsigargin for 24 h. Cells were incubated for 24 h in serum-complete medium. Luciferase production in cell lysates was measured by luminometry. Levels are expressed as light units (LU) per microgram of total protein. C282Y HFE decreased *bcl-2*-luc expression (**, *p *= 0.004) compared to WT HFE. Treatment with TUDCA significantly increased *bcl-2*-luc expression (#, *p *= 0.0238) compared to WT HFE. Z A1AT transfection significantly decreased *bcl-2*-luc expression **, *p *= 0.0029 compared to WT HFE transfected with M A1AT. Assays were performed in triplicate and are representative of at least three separate experiments. (*B*) HEK293 cells (1 × 10^6^) were transfected with C282Y HFE (M) with or without Z A1AT expression plasmids. Transfected cells were untreated or treated with 300 μM TUDCA, and cytosolic extracts were prepared 24 h posttransfection. (*B*) Ten-microgram of protein was assayed for cytochrome *c *release, and (*C*) caspase-3 activation by western blotting (*n *= 3). Incubation with TUDCA markedly inhibited cytochrome *c *release and processing of procaspse-3. (*D*) Blots were stripped and probed with β-Actin to confirm equal loading. (*E*) Measurement of caspase-3 activity in the lysates of HEK293 cells (1 × 10^6^) transfected with C282Y HFE (M) and either Z A1AT HFE or M A1AT expression plasmids. Transfected cells were untreated or treated with 300 μM TUDCA. Positive control of 2.5 μM thapsigargin used. Z A1AT increased C282Y HFE (M) caspase-3 activity compared to M A1AT (*, *p *= 0.0379) and treatment with 300 μM TUDCA significantly decreased C282Y HFE (M) induced caspase-3 activity compared to untreated (#, *p *= 0.0214).

C282Y HFE resulted in decreased expression of a *bcl-2*-promoter-linked luciferase reporter gene compared to WT HFE cells (** *p *= 0.004). TUDCA significantly increased *bcl-2 *luciferase activity in C282Y HFE-transfected cells treated with 300 μM TUDCA compared to untreated (Fig. 6A) (# *p *= 0.0238) (Fig. 6A). C282Y HFE significantly decreased *bcl-2 *promoter activity in the presence of Z A1AT compared to M A1AT (**, *p *= 0.0029) (Fig. 6A). WT HFE did not effect *bcl-2 *promoter activation in the presence of Z A1AT (Data not shown). To further elucidate both the anti-apoptotic effects of TUDCA and the effect of ZA1AT on C282Y HFE induced apoptosis, we examined cytochrome *c *release and caspase-3 activation by western immunoblotting in C282Y HFE transfected cells treated with 300 μM TUDCA and co-transfected with Z A1AT. In C282Y HFE cell lines treated with 300 μM TUDCA there was decreased cytochrome *c *release compared to untreated C282Y HFE cell lines. Furthermore, we found increased cytochrome *c *release in co-tranfected C282Y HFE and ZA1AT cells compared to C282Y HFE cell lines. Similarly, treatment with 300 μM TUDCA decreased this response (Fig. 6B). To further confirm this reduced apoptotic response by TUDCA we examined caspase-3 activity. We found that TUDCA significantly decreased caspase-3 activation in C282Y HFE cell lines treated with 300 μM TUDCA compared to untreated C282Y HFE cell lines. In addition, caspase-3 activation was increased in the co-transfected C282Y HFE and ZA1AT cell line compared to the C282Y HFE cell line. Treatment with 300 μM TUDCA decreased this response (Fig. 6C). Equal loading was confirmed by stripping the blots and probing with an antibody to β-Actin (Fig. 6D). We confirmed these findings by using a caspase-3 activity assay. In this assay HEK 293 cells were transfected with expression plasmids and thapsigarin was used as a positive control (Fig. 6E). We found that Z A1AT compared to M A1AT significantly increased caspase-3 activity in the C282Y HFE cell line (*, *p *= 0.0379). Finally, we found TUDCA significantly decreased caspase-3 activity in the C282Y HFE line, compared to untreated C282Y HFE cell line (#, *p *= 0.0214).

### C282Y stimulates IL-8 and MCP-1 protein production

**Figure 7:**
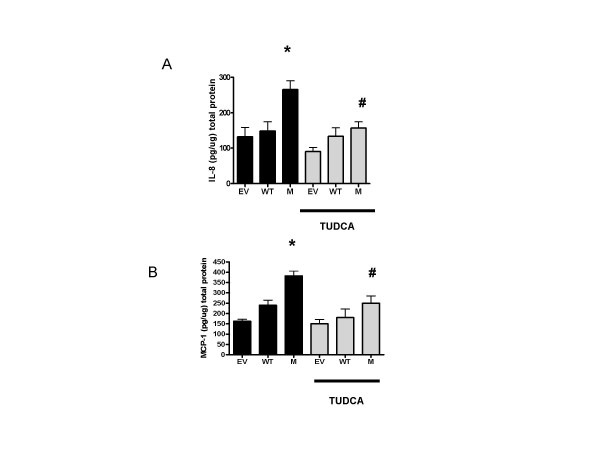
**C282Y HFE stimulates IL-8 and MCP-1 protein production**. C282Y HFE increases IL-8 and MCP-1 production. IL-8 (*A*) and MCP-1 (*B*) protein production was measured by ELISA form 24-h cell supernatants from HEK293 cells transfected with EV, N HFE, or C282Y HFE cDNAs with or without treatment of 300 μM TUDCA. Values are expressed as picograms per microgram of total protein. C282Y HFE increased IL-8 and MCP-1 protein expression (*, *p *= 0.0328 and *, *p *= 0.0149 respectively) compared to WT HFE. Treatment with TUDCA significantly decreased IL-8 and MCP-1 protein expression (#, *p *= 0.0235 and #, *p *= 0.0355 respectively) compared to WT HFE. Assays were performed in triplicate and are representative of at least three separate experiments.

To measure the effect of C282Y HFE protein overexpression on inflammation, MCP-1 and IL-8 protein production was measured in the transfected cell lines. Basal and C282Y-induced IL-8 and MCP-1 protein levels in cell supernatants from HEK 293 cells were quantified by ELISA (Fig. 7A and 7B). HEK cells produced a mean basal level of 40 ± 8 pg/μg of IL-8 protein and 30 ± 12 pg/μg of MCP-1. The actual concentration for the IL-8 and MCP-1 is shown after deduction of the HEK basal cytokine levels. *In vitro *transfection with EV increased basal IL-8 and MCP-1 expression due to the *in vitro *nature of this model system. However, WT HFE did not significantly increase either IL-8 or MCP-1 levels; whereas, C282Y HFE induced maximal IL-8 protein production from HEK 293 cells at 24 h (time-course experiments data not shown), increasing IL-8 levels to 260 ± 40 pg/μg of protein (*p *= 0.05) and MCP-1 levels to 380 ± 22 pg/μg of protein (*, *p *= 0.0328 and *, *p *= 0.0149 respectively compared to WT HFE transfected cells). Treatment of cell lines with TUDCA for 24 h significantly decreased IL-8 levels to 180 ± 30 pg/μg of protein and MCP-1 levels to 234 ± 40 pg/μg of protein (#, p = 0.0235 and #, p = 0.0355 respectively compared to WT HFE transfected cells).

## Discussion

In this study, a HEK293 cell line model system transiently expressing WT HFE or its C282Y mutant counterpart was used to determine the ER stress mechanisms associated with C282Y HFE production and retention. We demonstrated that the C282Y mutant protein co-localized with the ER resident protein calnexin and undergoes marked degradation compared to WT HFE, providing a suitable model of study for the C282Y HFE protein. C282Y HFE mutant protein accumulates in the ER and middle Golgi compartment, and consequently undergoes degradation [5,6,38-40]. Mutant proteins with such impaired egress have been shown to cause ER stress in a variety of disorders, which in turn may give rise to a variety of stress pathways such as EOR, UPR activation and cellular death [41-43].

Upregulation of the EOR pathway leads to activation of the transcription factor NF-κB [10]. In this study we found that C282Y HFE protein induced NF-κB, which consequently resulted in a marked increase in protein production of both interleukin-8 (IL-8) and monocyte chemotatic protein-1 (MCP-1), along with increased transcriptional activation of IL-8 in C282Y HFE-expressing cell line. IL-8 has been shown to be an important mediator in various diseases [44,45]. MCP-1 is secreted by a variety of cells as a response to several inflammatory stimuli and activates and attracts monocytes/macrophages [46]. Furthermore, MCP-1 concentrations are deregulated in patients with alcoholic hepatitis or cirrhosis [46,47] and in patients with hepatitis C [48]. These findings suggest that the pathology of HH in C282Y/C282Y HFE patients may involve an aberrant inflammatory action [49,50].

Indeed, in this regards Lee *et al *found that HFE expression was increased during ER stress, induced by serum deprivation, menadione and β-amyloid. This increase in HFE expression was independent of transferrin receptor and ferritin. Furthermore, the labile iron pool was consistently decreased when HFE expression was increased, suggesting that the observed induction of HFE has a protective function by limiting cellular iron exposure during stress [51,52].

UPR pathway involves up-regulation of the secretory pathway's capacity to process proteins and entails the transcriptional up-regulation of a co-ordinately expressed set of genes encoding ER chaperones, enzymes, and structural components of the ER. The UPR pathway culminates in the expression of glucose-responsive genes (*grp*) such as, *grp78*. Expression of the ER charpone *grp78*/BiP is a classical marker for UPR activation in mammalian cells. The *grp78 *promoter contains a consensus binding site called the ER stress response element; recognised by ATF6, a transcription factor specifically activated by ER stress. Several lines of evidence support the essential role of ATF6 in the ER stress response and have revealed it to be a proximal transducer of GRP78/BiP [14]. Recent studies of proteomic analysis of hepatic iron overload in mice have shown increased levels of GRP78/Bip protein [53]. This upregulation in response to iron excess has been demonstrated previously in human cells and it has been suggested that up-regulation of GRP78/Bip may indicate increased demand for re-folding or retention of proteins in the ER of iron-overloaded cells [54]. The UPR is also known to up-regulate CHOP, which is generally linked to ER stress. CHOP protein belongs to the CCAAT/enhancer-binding protein (C/EBP) family of transcription factors and is thought to play a critical role in cell survival or cell death during ER stress [55]. Prolonged activation of the UPR responses is known to result in cell death. Studies by Hacki et al, suggested that perturbing ER functions induces a specific crosstalk between the ER and mitochondria [56]. It is also worth noting that constitutive activation of NF-κB promotes survival of a range of cells, including B cells, hepatic cells and cancer cells. However, whereas NF-κB is most commonly involved in suppressing apoptosis by transactivating the expression of anti apoptotic genes, it is associated with promoting programmed cell death in response to ER stress *via *the EOR pathway by calcium release from the ER, resulting in mitochondrial dysfunction and apoptosis [57-59]. In neuronal degenerating disease, ER stress results in activation of NF-κB, up-regulation of GRP78 protein levels, and ensuing apoptotic cell death due to the expression of mutant protein [60]. The data in this study reveals a potential role for NF-κB in C282Y HFE mediated cellular death.

TUDCA is a non-toxic compound which is known to be effective in preventing cytotoxic processes. It has been demonstrated to inhibit activation of GRP78 and caspase-3, along with cytochrome *c *release. TUDCA stabilizes the lipid and protein structure of mitochondrial outer membranes, thus inhibiting Bax binding to the outer membrane [20]. Indeed, mitochondrial-derived reactive oxygen species may be involved [61,62] as suggested by recent studies which report that TUDCA prevents the generation of ROS [20]. The potential of TUDCA to prevent apoptosis and caspase activation may prove beneficial in protecting cells predisposed to disruptions in the ER. Cytochrome *c *release and caspase-3 activation along with decreased *bcl-2 *promoter activity suggest the damaging action of mutant C282Y protein in cellular stress and therefore TUDCA may protect cells during C282Y HFE protein induced stress.

A variable clinical presentation among C282Y homozygous individuals suggests an important contribution of disease modifiers acting on the same signalling pathways. Indeed, while the C282Y mutation results in an altered disease phenotype, mice studies have shown that the C282Y mutation does not completely disrupt the function of HFE, emphasising the importance of additional insults. Furthermore, activation of the UPR pathway can have a protective role [9]. Therefore, it may be suggested that activation of this pathway by C282Y HFE protein expression as shown in this report might explain the milder phenotype of C282Y HFE mice compared to a similar but more severe phenotype of the HFE knockout mice [63]. Z A1AT deficiency represents a possible genetic insult which is known to act on the EOR and UPR pathways and serves as an excellent model for conformational disease [64,65]. Recent work has revealed the lack of UPR activation by mutant Z A1AT, suggesting it is unlikely that mutant Z A1AT is "sensed" by the machinery of the UPR. Indeed, the signal appears not to be transmitted as a result of a block in the afferent or efferent components of the response, demonstrated by a lack of increased GRP78 activity. However, these studies have shown that Z A1AT UPR activation is found as a result of a secondary stimulus, revealing its activation in the presence of known chemical stress inducers [34]. We wanted to examine the co-existence of both C282Y HFE and Z A1AT in our *in vitro *cell model system, whereby the C282Y HFE could act as a genetic insult in the context of secondary stimuli acting on the Z A1AT UPR pathway. Our findings indicate the potential for C282Y HFE to activate the Z A1AT UPR pathway (Fig. 3A and 3D), thus implicating C282Y HFE as a factor which may 'trigger' and/or explain part of the clinical expression of Z A1AT deficiency and *vice versa*. Recent reports, have suggested that the heterozygous carrier state for the mutant Z gene, found in 1.5 % to 3% of the population, is not itself a common cause of liver injury but may be a modifier gene for other liver diseases [27]. Indeed, a large patient population study recently indicated that the Z A1AT heterozygous state may have a role in worsening liver disease [66].

## Conclusion

This study examines the effect of C282Y HFE protein expression upon specific ER stress pathways. It is possible that differences in the quality control systems within the ER, could explain some of the phenotypic variation observed in HH. To test this hypothesis there is a need to understand how C282Y HFE protein expression interacts with the ER quality control components. While the results from our study are interesting and novel, potential limitations in regards applicability of our findings to the human condition must be considered. Our observations might be influenced by the magnitude of expression of the mutant protein, the cell line chosen for the studies and the relative magnitude of β_2_M expression. Nonetheless, these findings of intracellular stress mechanisms associated with C282Y HFE raises the prospect of a better understanding of the pathogenesis of HH and as a model system may serve as a platform to test the effects of the UPR on proteins involved in iron metabolism.

## Methods

### C282Y HFE model system

Human Embryonic Kidney (HEK) 293 cells (American Type Culture Collection, Manassas, VA) were cultured in Eagle's Minimum Essential Medium supplemented with 2 MM L-glutamine, 1% non-essential amino acids, 1% sodium pyruvate and 10% foetal calf serum (Invitrogen, Scotland). HEK 293 cells were transiently transfected with eukaryotic expression vectors containing a normal HFE or mutant C282Y cDNA. The cDNA sequence obtained from GenBank (U60319) was assembled using synthetic oligonucleotides (Geneart, Regensburg, Germany).

The fragment was cloned into pcDNA3.1 (Invitrogen) using BamHI and NotI restriction sites with or without the cDNA for green fluorescent protein or His tag to generate plasmids expressing fluorescent-tagged (GFP) or His tag versions of wild-type (WT) HFE and C282Y HFE (M). The plasmid DNA was purified (Pure Yield™ Plasmid Midiprep, Promega) from transformed Escherichia coli. The C282Y mutation was inserted utilizing QuickChange site-directed mutagenesis kit (Stratagene) with mutagenic primers encoding the appropriate G to A base change. The final constructs were verified by sequencing. The M A1AT and Z A1AT vectors were made whereby the M A1AT cDNA was cloned on a 1256-bp *Eco*RI/*Xho*I fragment into pZeoSV2^+ ^(Invitrogen, Carlsbad, CA) to generate pMA1AT. Z A1AT was constructed by site-directed mutagenesis using the QuikChange site-directed mutagenesis kit (Stratagene) and mutagenic primers encoding the Glu^342 ^→ Lys mutation (bold) and a *Cla*I restriction site (underlined) for screening (forward primer, 5'-GCT GTG CTG ACC ATC GAT**A**AG AAA GGG ACT GAA GCT GCT G-3'). Putative mutants were isolated, and their sequences were verified.

### Transfection

HEK293 cells were seeded at 1 × 10^6 ^on six-well plates or 6 × 10^6 ^in flasks 24 h before transfection. Transfections were performed with GeneJuice transfection reagent (Novagen) in a 1:1 ratio according to the manufacturer's instructions for 1 h with 1 μg pcDNA3.1 (empty vector), pWT HFE, or pC282YHFE with or without 1 μg pZeoSv2^+ ^(empty vector), pMA1AT, or pZA1AT. After supplementation with complete medium, cells were incubated for 24 hours. Uniform transfection efficiencies were verified by measuring β-galactosidase activity using the substrate *o*-nitrophenyl-β-D-galactopyranoside from a cotransfected β-galactosidase expression plasmid.

### Confocal Microscopy

Cells for confocal microscopy were grown on chamber slides (Labtek) and transfected with green fluorescent-tagged versions of WT HFE and C282Y HFE as described above. At 8, 16 and 24 h after transfection, cells were washed, fixed with paraformaldehyde for 30 min at room temperature, washed again, and blocked for 60 min with phosphate-buffered saline (PBS) containing 10% bovine serum albumin, 0.1% Triton X-100, and 0.1% sodium azide, and then incubated in primary antibody goat polyclonal IgG calnexin (Santa Cruz Biotechnology) for 90 min. Cells were washed again, incubated with secondary to goat IgG Texas Red (Abcam) for 60 min, washed, and mounted in Fluorescent mounting medium (DakoCytomation, CA, U.S.A) containing 2% 1,4-diazabicyclo [2.2.2]octane (Sigma) and 3 μg/ml 4',6-diamidino-2-phenylindole (Sigma). Pictures were taken using the FV1000 Olympus confocal laser microscope.

### C282Y HFE protein production measurement by sandwich ELISA

C282Y HFE-His tag protein production in lysates was measured by sandwich ELISA using mouse monoclonal IgG Penta-His (Qiagen), rabbit anti-mouse IgG (Qiagen, UK), goat anti-rabbit IgG HRP (DAKO, Carpenteria, CA), and ABTS substrate (Zymed Laboratories, San Francisco, CA). After absorbance reading at 405 nm on a multilabel counter (Victor2; Wallac, Gaithersburg, MD), C282Y HFE-His tag protein levels were calculated from both an Multiple tag cell lysate (GenScript Corporation, NJ) and 6 × His protein ladder (Qiagen, UK) standard curve. Values are expressed as nanograms per 6 × 10^6 ^cells.

### Reporter gene assays

HEK cells were seeded at 1 × 10^6 ^cells/well and were cotransfected with 1 μg of pcDNA3.1 (empty vector), pWTHFE, or pC282YHFE; with or without 1 μg pZeoSv2^+ ^(empty vector), pMA1AT, or pZA1AT and either 1 μg of *grp78 *promoter-linked luciferase plasmid (a gift from K. Mori (Laboratory of Molecular Neurobiology, Graduate School of Biostudies, Kyoto University, Kyoto, Japan)) [12], or *ATF6 *promoter-linked luciferase plasmid (a gift from R. Prywes (Department of Biological Sciences, Columbia University, New York, U.S.A)) [14] or *CHOP *promoter-linked luciferase plasmid (a gift from P. Fafournoux (Unité de Nutrition Cellulaire et Moléulaire, INRA de Theix, Champanelle, France) [35] or *bcl-2 *a gift from L. Boxer (Center for Molecular Biology in Medicine, Department of Medicine, Stanford University School of Medicine, Stanford, California, U.S.A) [36], or *IL-8 *luciferase reporter gene plasmid or an (NF-κB)_5_-luciferase reporter gene plasmid [34]. Transfections were incubated for 1 h at 37°C. Cells were then supplemented with additional growth medium (4 ml/well) for 24 h at 37°C before being left untreated or treated with 2.5 μM thapsigargin or 200 μM/300 μM tauroursodeoxycholic acid for 24 h. Cells were lysed with reporter lysis buffer (Promega; 250 μl/well), protein concentrations were determined [37], and reporter gene activity was quantified by luminometry (Victor 1420 multilabel counter; Wallac) using the Promega luciferase assay system according to the manufacturer's instructions. Reporter gene expression was expressed as light units per microgram of total protein.

### Western blot analysis

Cell cytoplasmic extracts (10 μg of protein) were separated by electrophoresis on 10% SDS-polyacrylamide gels and transferred to a nitrocellulose membrane (Sigma-Aldrich) in 20 mM Tris, 150 mM glycine, 0.01% SDS, and 20% (v/v) methanol at 75 mA for 2 h using a semidry electrophoretic blotting system. Nonspecific binding was blocked with 0.2% I-Block (Tropix, Bedford, MA) and PBS containing 0.1% Tween 20 (Sigma-Aldrich). Immunoreactive proteins were detected by incubating the membrane with a specific Ab (GRP78 (N-20) (from Santa Cruz Biotechnology); mouse anti-human IκBα (from Cell Signaling Technologies, Beverly, MA); Mouse anti-human cytochrome *c *(from BD Biosciences); Rabbit anti-human caspase 3 (from Affiniti Research Products Ltd.); mouse anti-actin (Actin (Ab-1) Kit) (from Oncogene Research Products). After six 5-min washes with PBS containing 0.1% Tween 20, immunoreactive proteins were detected using an appropriate alkaline phosphatase-conjugated secondary Ab (Promega) and CDP-Star chemiluminescent substrate solution (Sigma-Aldrich) according to the manufacturer's instructions.

### Caspase-3 activity assays

Caspase-3 activity was measured using the flourogenic substrate Ac-DEVD-AMC (Caspase-3 Substrate II, Calbiochem). Cells were lysed in 250 μl of 25 mM Hepes, 5 MM edta, 0.1% Chaps, 5 mM ATP, pH 7.5, with 2 mM DTT. Equal amounts of protein from each sample were incubated with substrate (50 μM, final concentration) in lysis buffer for 30 min at 37°C, and fluorescence (substrate turnover) was determined by excitation at 355 nm and emission at 460 nm. Activity is expressed as Δ fluorescence units (dFU)/μg protein. Assays were performed in triplicate.

### IL-8 and MCP-1 protein production

The human cell line HEK293 was transfected as already described. Cells were then left untreated or treated with 300 μM tauroursodeoxycholic acid for 24 h. IL-8 and MCP-1 protein concentrations in cell supernatants were determined by ELISA (R&D Systems, Minneapolis, MN). Protein concentrations were determined by the method of Bradford [37]. Cell viability, assessed by trypan blue exclusion, was >95% in all studies. Results are expressed as picograms per microgram of total protein.

### Statistical analysis

Data were analyzed with the PRISM 4.0 software package (GraphPad, San Diego, CA). Results are expressed as the mean ± SE, and were compared by *t *test or ANOVA as appropriate. Differences were considered significant when the p value was ≤ 0.05.

## Authors' contributions

MWL conceived of the study, and participated in its design, carried out molecular analyses and drafted the manuscript.

AKM carried out the immunoassays and helped to draft the manuscript.

MW participated in molecular analysis.

MJOD participated in molecular analysis.

SN participated in design of study and co-ordination and helped to draft the manuscript.

All authors have read and approved the final manuscript.
